# Community pharmacist counseling in early pregnancy—Results from the SafeStart feasibility study

**DOI:** 10.1371/journal.pone.0219424

**Published:** 2019-07-19

**Authors:** Maria Bich-Thuy Truong, Elin Ngo, Hilde Ariansen, Ross T. Tsuyuki, Hedvig Nordeng

**Affiliations:** 1 Department of Pharmacy, University of Oslo, Oslo, Norway; 2 The Norwegian Pharmacy Association, Oslo, Norway; 3 Department of Pharmacology, University of Alberta, Edmonton, Canada; 4 Department of Child Health and Development, Norwegian Institute of Public Health, Oslo, Norway; University of Wisconsin Madison School of Pharmacy, UNITED STATES

## Abstract

**Background:**

Community pharmacists are available to counsel women in early pregnancy, but no studies have assessed the feasibility of such a service.

**Objective:**

To test the feasibility of a pharmacist consultation in early pregnancy and to inform the design of a definitive trial.

**Setting:**

Six community pharmacies in Norway from Oct. to Dec. 2017.

**Method:**

We evaluated recruitment approaches and an automatic data preprocessing system (ADPS) to enroll, assign participants, and distribute questionnaires. Women (≥18 years) in early pregnancy were eligible for inclusion. Participants were assigned to a pharmacist consultation (intervention group) or standard care (control group). The intervention aimed to address each woman’s concerns and needs regarding medications and ailments in pregnancy, and was documented on a standard form. The women’s acceptability of the intervention was measured by a questionnaire.

**Main outcome measures:**

Appropriate recruitment approaches, workflow of the ADPS, and women’s acceptability of the intervention.

**Results:**

Of the 35 participants recruited, 19 were recruited through Facebook. The ADPS worked well. Treatment of nausea and vomiting (NVP) (10/11) and general information about medications (8/11) were frequently discussed during the consultations (n = 11). The women reported high satisfaction with the consultation. Having the option of telephone and follow-up consultations was important to the women.

**Conclusion:**

It is feasible to provide community pharmacist consultations in early pregnancy. In a definitive study, the consultations should focus on NVP and general medication use and further explore social media as a recruiting tool. Both in-pharmacy and telephone consultations should be offered to deliver the intervention.

## Introduction

Up to 60–80% of pregnant women use at least one medication [1–3], and more than half have perceived needs for information about the safety and use of medications during pregnancy [4, 5]. Studies have also shown that pregnant women tend to overestimate the teratogenic risk of medications [6, 7], often resulting in unfound anxiety [7], non-adherence to needed medications [8, 9], and the use of herbal or “natural” medications [10]. These findings highlight the necessity to address pregnant women’s individual information needs and empower them to make informed treatment decisions during pregnancy to ensure maternal and fetal health.

The patient-centered approach is a growing expectation in antenatal care [4], and pregnant women express that they want to be actively involved in choosing the course of treatment during pregnancy [11]. However, they have also stated that they do not receive adequate counseling from their healthcare providers [12] and subsequently seek health information from other sources, e.g. the internet [5, 13]. Enhanced communication between pregnant women and their healthcare providers is fundamental to promote women’s health during gestation. Discussing the benefits and risks associated with taking, not taking, stopping, or altering dosages of medications while pregnant has previously been described as vital to enabling pregnant women to make informed treatment decisions [11]. Such counseling should be provided as soon as possible in pregnancy or ideally pre-conception, to optimize treatment from the very beginning of the pregnancy. Unfortunately, in many countries there seems to be a gap in antenatal care from conception until the first consultation, which is often at the end of the first trimester [14, 15].

Community pharmacists have been described to have an extensive role in the medication counseling of pregnant women because of their accessibility in the community, as well as their specialized training in pharmacotherapy and pharmaceutical care [16]. Pregnant women also consider community pharmacists as a trusted medication information source [5]. Community pharmacy services providing counseling, support, and information regarding medication use have shown positive effects on medication adherence and other clinical outcomes in several other patient populations [17]; yet, pharmacist care is not part of the routine antenatal care for pregnant women.

## Aim of the study

The overall aim of this study was to test the feasibility of performing a randomized controlled trial (RCT) of a structured and standardized pharmacist consultation in early pregnancy, and to inform the design of a future definitive trial. The more specific aims were to: 1) test several recruitment strategies, 2) estimate recruitment and attrition rates, 3) describe the timeframe and content of the consultation, 4) assess patients’ acceptability of the intervention, and 5) test an automatic data preprocessing system (ADPS) to enroll participants, assign them to one of the two study groups, and distribute questionnaires to them.

## Methods

### Design, setting, and study pharmacists

This feasibility study was conducted as an assigned intervention study. The study was conducted from October to December 2017 in six community pharmacies by four study pharmacists in Norway. All study pharmacies were located in urban areas limited to the South-Eastern region. As this was a feasibility study, the study pharmacists were selected based on convenience. All study pharmacists completed a training program including three e-courses in pharmacotherapy in pregnancy and self-study of a compendium covering common pregnancy-related ailments. The e-courses were developed by the Centre for Competence and Development for Pharmacies (Apokus) for community pharmacists, while the compendium was developed by the project team. The topics covered during the training was common pregnancy-related ailments, e.g., nausea and vomiting, heartburn and reflux problems, pain and headache, constipation, nasal congestion, and common cold, in addition to medication use for asthma, allergy, diabetes, epilepsy, infections, and during breastfeeding.

### Participants and sample size

All Norwegian speaking pregnant women in their first trimester (≥18 years) were eligible for inclusion. A sample size of 5 to 20 participants is usually reasonable within the scope of feasibility testing [18]. Our sample size was determined based on these sample size recommendations taking into account that this is the first time a potential community pharmacy service for pregnant women will be tested out [18, 19]. We aimed therefore to recruit a total sample of 35 participants allowing for a 40% dropout/lost to follow-up.

### Recruiting strategies

#### Social media

The recruiting of participants through the study’s Facebook page included both organic posts and promotions of selected posts. The organic posts were published daily throughout the study period. The promotions were set to target women between the age of 18 and 40 years, with interests within the field of “pregnancy” and residence in the areas of the six study pharmacies. The same promotions were also distributed from the study’s Instagram profile. The evaluation of the social media recruiting was based on the number of participants enrolled in the study via this method.

#### Other approaches to recruiting

Other approaches to recruiting included features in several websites relevant for pregnant women, and posters and flyers in the local areas of the six study pharmacies. The websites were forums for pregnant women, including altformamma.no (“allformommy”), libero.no, babyverden.no (“babyworld”), and the teratology information service for the public, tryggmammamedisin.no (“safemommymedicine”). The evaluation of these approaches was measured by the number of enrollments as a direct result of the features, posters, and flyers.

### Study webpage and online consent form

All recruiting methods referred the participants to the study webpage for a complete study description and access to the consent form [20]. The online consent form required login with an electronic ID twice to meet the Data Privacy legislation and hence ensure that the identity of the consent-giver. The number of non-completed consent forms (login once only) was recorded.

### Baseline questionnaire (Q1)

An email with link to the baseline questionnaire (Q1) was sent to all participants immediately after study enrollment. The Q1 included questions about where subjects first saw information about the study (used to calculate enrollment numbers according to recruitment strategies); sociodemographic factors (age, area of residence, occupation); pregnancy-related factors (gestational age and parity); and nausea and vomiting in pregnancy (NVP) severity by the Pregnancy-Unique-Quantification-of-Emesis 24 (PUQE-24) scale [21]. Data are summarized in Table 1. The PUQE-24 is a three item questionnaire with a score of 1–5 on each item. The score ranges from 3–15, where a score ≤6 is categorized as mild, 7–13 as moderate, and ≥13 as severe. The women were also asked to state their general wellbeing using a score from 0 to 10, with 0 being “worst possible” and 10 being “the best wellbeing compared to the pre-pregnancy state” as part of the NVP severity assessment [21].

**Table 1:** Sociodemographic and pregnancy-related factors of the participants at baseline.

Sociodemographic factors	n = 30
**Age**	
≤25	3
26–30	11
31–35	11
≥36	5
**Residence according to the Norwegian health regions**	
Central	0
Northern	0
South-Eastern	25
Western	5
**Occupation**	
Healthcare personnel	10
Employed in other sectors	15
Other	5
**Pregnancy-related factors**	
**Gestational age at recruitment**	
Median week (IQR)	9 (7–11)
**Primiparous**	
Yes	13
No	17
**Nausea and vomiting in pregnancy (NVP)**	
Women with NVP	23
PUQE-score, median (IQR)	7 (5–9)
Women with PUQE ≥7^a^	16
Wellbeing, median (IQR)^b^	8 (5–9)
**Other pregnancy-related ailments**^c^	
Cold/nasal congestion	15
Constipation	14
Headache	11
Heartburn and reflux problems	10
Sleeping problems	9
Pain in the back, neck, or pelvic girdle	9
Other	5
Dizziness	2
Diarrhea	2
None	2

IQR, Interquartile range; PUQE, Pregnancy-Unique Quantification of Emesis.

^a^PUQE-score ≥7 equals moderate or severe NVP.

^b^0 = worst possible, 10 = as good as you felt before pregnancy, n = 18.

^c^The total number does not add up as a woman could report several pregnancy-related ailments; “Other” includes: eczema, flatulence, acne, breast tenderness, and fatigue.

The participants were presented with a list of eight common pregnancy-related ailments (i.e., common cold or nasal congestion, constipation, headache, heartburn and reflux problems, NVP, pain in the neck, back or pelvic girdle, sleeping problems, and urinary tract infections). Other ailments not listed could be entered as free text. For each reported ailment, the participants were asked to report any related medication use (i.e., name of medication and timing of use during pregnancy).

### Assignment to the study groups and selection of study pharmacy

Based on the timing of enrollment, every second participant was assigned to the intervention group. An email with a link to the booking form was sent to participants in the intervention group 20 minutes after enrollment. The booking form allowed the participants to suggest a date and time for their consultation, as well as which of the six study pharmacies they preferred to have the consultation performed at. Other inquires could be entered as free text. The preferred pharmacist contacted the participants either by SMS or by telephone to confirm the booking or to suggest another date and/or time. Participants in the control group received an email with general information about the study.

### The intervention

The intervention was carried out in the pharmacies’ information rooms or over the telephone for a duration up to 15 minutes. The intervention was defined as “*A planned*, *structured*, *and individualized consultation with the purpose to relieve pregnant women for any concern and answer questions she may have regarding ailments and medication use during pregnancy”*. The study pharmacists had access to each participant’s answers from the Q1 before the consultation. The study pharmacist documented each consultation on a standard form, e.g., time spent on preparatory work, on the actual consultation, on writing up the summary of the consultation, in addition to the content. The need for follow-up consultations was also assessed by the study pharmacists using clinical judgment. Immediately after the consultation, the participants were asked to complete a satisfaction questionnaire (Table 2).

**Table 2:** The participants in the intervention group (n = 11) were asked to evaluate the intervention by answering these questions.

Question	Answer, total n = 8
**Did you find it useful to speak to your pharmacist about medications and ailments during pregnancy?**	Median score: 5.0 (range: 3–6)
*Score*: *0–6; 0 = not useful at all*, *and 6 = very useful*	
**In what way was it useful?**	*“I could ask necessary questions*, *and get answers that have helped me later”* “I didn’t know that I can use xylometazoline during pregnancy” “*I got concrete and useful information”* “To get confirmation that it is ok to use medications prescribed by doctors during pregnancy”
*Optional free text entry*	
**To which degree did you get the information you were seeking?**	Median score: 5.0 (range: 2–6)
*Score*: *0–6; 0 = “No new information*, *I didn’t get the answers I was looking for”*, *and 6 = “A lot of new information*, *I got all the answers I was looking for”*	
**Is there anything that could improve your benefit from this consultation?**	“Written materials and maybe another consultation later in pregnancy” By a women who received the consultation by telephone: *“A consultation in person”*
*Optional free text entry*	
**Would you recommend this consultation to other pregnant women?**	
Yes	7
No	1
**Do you think this consultation should be offered to all pregnant women?**	
Yes	6
No/I don’t know	2
**If yes, who should cover the expenses?**	
The government as part of antenatal care	5
Pregnant women themselves	1
The pharmacies	0

A consultation guide was provided to the study pharmacists to structure the consultation. The guide divided the consultation in to three parts; the introduction, the mid-part, and the closing (S1 File) [22, 23].

### Technical procedures

**Fig 1:**
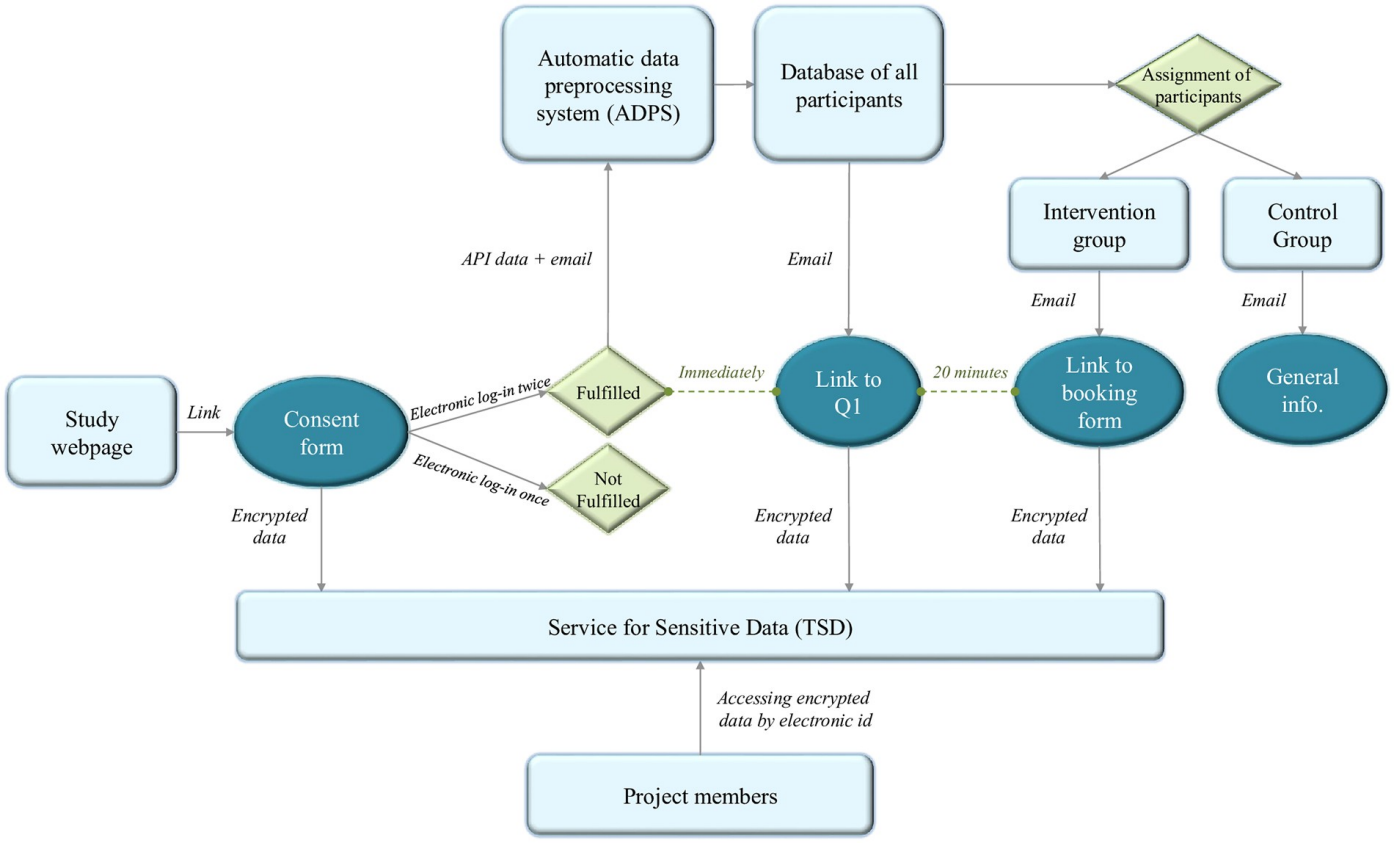
Overview of workflow. Workflow of the technical procedures for consent, assignment of participants to one of the two study groups, and the distribution of emails in the study. **API**: Application programming interface. **Q1**: Baseline questionnaire.

The enrollment of participants, distribution of emails, and assignment of participants were automatically done using the ADPS. The system identified participants who had fulfilled the two logins for the online consent form, distributed the Q1 by email, assigned all participants to one of the two study groups, and distributed the booking form and the general email to participants assigned to the intervention and the control groups, respectively (Fig 1). Reminders to complete the Q1 and respond to the booking form were also sent automatically by email three days after enrollment based on whether or not the participant had clicked on the link.

#### Data storage

All data collected were stored at the University of Oslo’s Service for Sensitive Data (TSD). The TSD is designed for storing and post-processing of sensitive data in compliance with the Norwegian Personal Data Act and Health Research Act.

### Attrition rates

The numbers of women failing to respond to the Q1, book the intervention, and/or complete the intervention were recorded. The women could at any time dropout of the study either by contacting the research group or by the link provided in each e-mail. The link directed the participants to a simple questionnaire asking whether or not they were willing to state a reason for dropout. The alternatives were: “I am no longer pregnant”, “The questionnaires require too much time”, “I was allocated to the control group”, “The nearest study pharmacy was too far away”, “Other”, and “I prefer not to give a reason for dropout”. Non response without filling out the dropout questionnaire was considered “Lost to follow-up”.

### Analysis

All descriptive statistics, e.g. number of cases, median values, standard deviation, minimum and maximum (range), and inter quartile range (IQR), were obtained using the “Tabulation” and “Summarize” function in StataSE version 15. Only complete cases were analyzed.

### Ethical approval

This study was approved by the Regional Committees for Medical and Health Research Ethics in Norway (Ref: 2016/1686). Informed consent to participate in the study was obtained from all participants.

## Results

### Study sample

**Fig 2:**
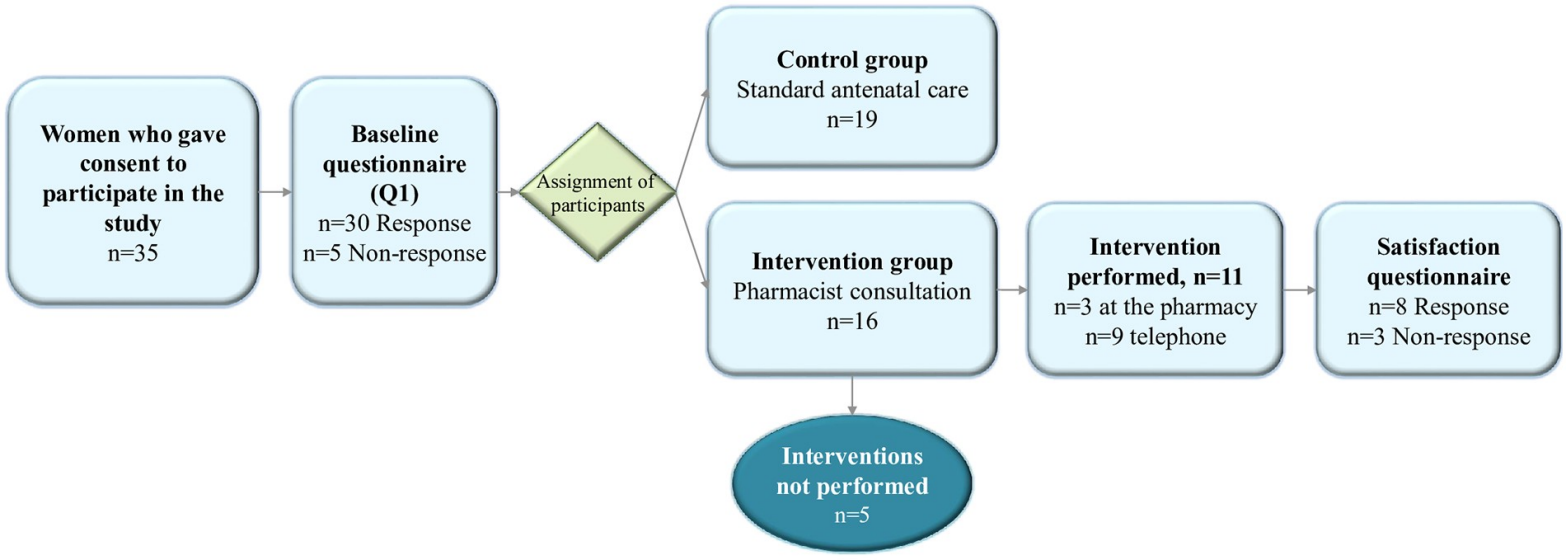
Flowchart. Flowchart of the allocation of participants in the two study groups, response rate of baseline questionnaire, and number of interventions performed.

In total, 35 participants completed the online consent form and enrolled in the study. The participants’ average age was 30.6±5.1 years (range: 23–47 years) and the median gestation stage at recruitment was 9 weeks (IQR: 7–11 weeks). The majority of participants (28/30) had experienced at least one pregnancy-related ailment, and 23/28 of these participants had experienced NVP. The majority were employed (25/30) and about half were primiparous (13/30). Sociodemographic and pregnancy characteristics for the women in the study sample are presented in Table 1. Sixteen participants were assigned to the intervention group and the remaining 19 were in the control group (Fig 2).

### Recruitment

The participants were largely recruited through social media, with Facebook and Instagram yielding 19 and 1 participant, respectively. The remaining participants were recruited through friends and families (5/30), other online publicity (3/30), and with posters and flyers (2/30).

### The consultation

#### Timeframe

The four study pharmacists performed one to five consultations each. The median duration of the interventions (n = 11) was 15 minutes (IQR: 12–20 minutes). The median preparation time by the pharmacist before each consultation was 30 minutes (IQR: 10–60 minutes). The preparations typically consisted of making an appointment with the participant and reading through the baseline characteristics.

#### Women’s concerns and needs

The majority of women (10/11) reported that they wanted general information about the management and treatment of common pregnancy-related ailments. Specifically, they asked for information regarding NVP (n = 10), nasal congestion (n = 5), headache (n = 4), constipation (n = 3), sleeping problems (n = 3), reflux and/or heartburn (n = 2), pain in the back (n = 1), and diarrhea (n = 1).

The second most discussed topic was general medication use during pregnancy (n = 8). The medications discussed were typically over the counter (OTC) medications, e.g., paracetamol (n = 4), xylometazoline (n = 3), and ibuprofen (n = 2). Two women also wanted to discuss the use of metoclopramide and meclizine for NVP. The safety effects of paracetamol on the unborn child were also a main topic in two consultations. One woman was advised to see her general practitioner due to pain related to Braxton Hicks contractions early in pregnancy.

#### Other aspects of the consultation

Seven women inquired about having the consultation over the telephone due to residency far from the six study pharmacies. An additional two consultations were performed over the telephone due to severe NVP symptoms of the participants. The pharmacists scheduled five follow-up consultations in total, mostly to follow-up on treatment advice for NVP and heartburn.

### Patient acceptability

Eight satisfaction forms were filled out by the women in the intervention group (8/11). These indicated that the women found the consultation very useful (median satisfaction score 5, range: 3–6), and that the majority of women (7/8) would recommend the consultation to other pregnant women. The free text entries revealed that the women found it most useful to get information tailored to their needs (Table 2).

### Attrition rates

Five participants did not respond to the Q1, and five interventions were not performed. Four of five women who did not book the intervention dropped out of the study due to pregnancy loss. The total attrition rate was 10/35 (not including the satisfaction form).

### Technical procedures

Five participants did not fulfill the online consent form process by logging in twice consecutively. Four of these women did enroll in the study after receiving a reminder by email. The automatic assignment of the participants was modified and improved several times during the study period. The modifications were related to improving the technical automatization of the ADPS. This resulted in an uneven number of participants in the two study groups. The ADPS worked well to enroll, assign participants to one of the two study groups, and distribute emails.

## Discussion

Community pharmacists can have an important role in the medication counseling of pregnant women [16]. To the best of our knowledge, this is the first study investigating the feasibility of providing a structured and standardized community pharmacist consultation for pregnant women in early pregnancy. This study showed that recruitment of pregnant women was feasible, that the pregnant women’s satisfaction with the consultation was high, and the results provided important information for a future definitive RCT.

### Recruitment

Social media was the strategy that yielded the most participants (20/35), but recruiting took longer than initially expected. Throughout the study period of three months, the average recruitment rate was 11 participants per month. Inconvenience and randomization have been described as barriers for pregnant women to participate in clinical research [24–26]. Fear of potential risks, e.g., in more invasive studies, or apprehension to take medications during pregnancy has also been identified as barriers [26]. For our study, lack of knowledge about the pharmacist’ expertise may have prevented women from participating. Recruitment through social media, especially Facebook, should be further exploited as it can be useful in health research by decreasing the recruitment period [25]. Moreover, social media recruiting of pregnant women can be more feasible and inexpensive than in-person methods [27], as well as more effective in reaching women earlier in pregnancy than traditional methods [28]. The social media approach may also be beneficial to reach women that do generally not have regular contact with the healthcare system. Pharmacists can remind them of the importance of early care and monitoring, and possibly facilitate the connection with appropriate healthcare providers, e.g., physician or midwife.

### Attrition

A future study needs to consider a relatively high level of attrition. As the proposed pharmacist consultation specifically targets pregnant women in their first trimester, attrition due to spontaneous abortion should be included in the sample size and power calculation in a full-scale study. Up to 30% of all pregnancies end in spontaneous loss during the first 12 gestational weeks [29]. Therefore, it was not surprising, nor avoidable, that pregnancy loss was the major reason for dropout in this feasibility study. The trade-off between a higher risk of attrition and recruiting later in pregnancy was discussed. However, as the potential benefit of a pharmacist consultation may be greatest in early pregnancy, when the gap in antenatal care occurs, we recommend that a future full-scale study continues to recruit and offer the consultation to pregnant women in their first trimester.

In total, 5/35 participants did not respond to the baseline questionnaire Q1. No measures were used to increase response rates beside a reminder by email after three days. To minimize the issue of selection bias due to non-response, it is important to consider including incentives to encourage pregnant women to participate in studies. Conditional lottery tickets and monetary incentives have been successful in increasing response rates to online questionnaires and retention in randomized trials [30]. Another possibility is to emphasize the importance of retention and how the participants can make valuable contributions to improve care for pregnant women in the Patient Information Leaflet [31].

### The intervention

The results from this feasibility study indicate that 15 minutes per consultation seems reasonable and feasible. Although, only 11 consultations were performed; therefore, these results should be interpreted with caution. However, the timeframe of 15 minutes is in line with other patient-centered pharmacy services, e.g. the New Medicine Service and the Medicine Use Review in England (10–15 and 10–20 minutes, respectively) [32].

The prevalence of NVP in our study sample, reported by over three quarters of women, was in line with what was expected from population prevalence rates [33]. Considering the high prevalence of NVP and that most pregnant woman do not have established contact with healthcare personnel when the symptoms occur, the pharmacist consultation should specifically target this condition. The NVP focus is also appropriate from a pharmacist’s point of view [34]. The pharmacist can identify women suffering from NVP and assess the severity by utilizing the easy-to-use PUQE-24 scale [21]. Women with mild NVP can be advised about lifestyle and diet changes that may improve symptoms. Women with moderate to severe symptoms can be referred to their general physician to further assess the need for pharmacologic treatment. These measures seem applicable in an everyday pharmacy setting without substantial changes.

The general use of medications, with a focus on OTC medications, was also frequently discussed during the consultations. To focus the pharmacist consultation on OTC medications is reasonable as this is within the core expertise of a community pharmacist. Many women had concerns regarding medication use during pregnancy independent of if they were currently using the medications or not. The majority of women had this precautionary need for information.

Moreover, as convenience has been described as one of the main facilitators for pregnant women to participate in clinical research [26], we would highly recommend that a future definitive trial provide the pharmacist consultation over the telephone in addition to the in-pharmacy consultation. The telephone counseling was not initially planned in our feasibility study but was offered as many participants specifically requested this.

### Patient acceptability

The participants who received the consultation reported high satisfaction with the service. They specifically pointed out that the possibility to get information tailored to their situation was highly appreciated. This is in line with previous studies suggesting that women want to be involved in decisions about treatment during pregnancy [11]. However, as we only received eight (8/11) satisfaction questionnaires, the results may not be generalized to all pregnant women and should be interpreted with caution.

### Limitations

The main limitation of this feasibility study is the low number of participants (n = 35) and women who completed the satisfaction questionnaire (n = 8). As the main topics and trends were clear, we do not expect that increasing the number of participants would materially change our conclusion. Moreover, the pharmacists’ fidelity to the intervention was not formally evaluated. All study pharmacists, however, received a protocol with a description of how the consultation should be performed and the project members were available for further questions throughout the study period. The informal feedback from the pharmacists was that having information from the Q1 before the consultation was highly valuable as they could prepare. The pharmacists’ feedback should be collected in a structured manner to further inform the study design of a subsequent RCT. Lastly, as this feasibility study was carried out in Norway, we do not know how generalizable our findings are to other countries with different healthcare systems and maternity care schemes. The role of the pharmacist also varies in different countries and this may impact the results of a feasibility study.

### Patient outcomes in a future trial

A future definite trial investigating the effect of a pharmacist consultation for pregnant women in early pregnancy should ensure that data on health outcomes, medication use, and utilization of health care services in pregnancy are collected. This will ensure that also the cost effectiveness of the intervention can be assessed.

## Conclusion

This feasibility study demonstrated that a RCT of a pharmacist intervention for pregnant women in early pregnancy is achievable. A full-scale study should have the pharmacist consultation focus on NVP and general medication use, carefully consider drop-outs due to pregnancy loss, and further explore social media as a recruiting tool. Both telephone and in-pharmacy consultations, as well as follow-ups, should be offered as part of the service. Though the logistics worked well, further testing should be done on a larger scale to identify the benefits and actual resources needed to establish such a service.

## Supporting information

S1 File Questionnaires. Questionnaires used in this feasibility study, including the baseline questionnaire, the booking form, and the dropout form.
(DOCX)

S2 File Consultation guide. (DOCX)
