# Supplementary material for: Community pharmacist counseling in early pregnancy—Results from the SafeStart feasibility study
Source: PLoS One. 2019 Jul 19;14(7):e0219424. doi: 10.1371/journal.pone.0219424 (PMC6641474; doi:10.1371/journal.pone.0219424)
Supplement: S1 File — Questionnaires used in this feasibility study, including the baseline questionnaire, the booking form, and the dropout form. (DOCX) [file pone.0219424.s001.docx]

| University of oslo |
| --- |
| The SafeStart Feasibility Study |
| English questionnaires |
| Baseline Q1  Booking form  Drop out form |
|  |
|  |

|  |
| --- |

**General INFORMATION**

| 1. **In which pregnancy week are you in now (0-12)?** |
| --- |
| 1. **Where did you first see information about this study?** |
| 1. **In what county do you live?** |
| 1. **What is your birth year?** |
| 1. **Have you been pregnant before?**   □ No, this is my first pregnancy  □ Yes |
| 1. **What is your occupational status?**   □ Student  □ Employed  □ Other |

| **PREGNANCY-RELATED AILMENTS**   \| 1. **Have you experienced any pregnancy-related ailments since the start of your pregnancy?** Examples of ailments are nausea and vomiting in pregnancy, heartburn, reflux problems, constipation, common cold etc.   □ Yes  □ No \| \| --- \| \| *If “yes”:* **Which of these ailments have you experienced?** You can tick off for several ailments.  □ Nausea and vomiting  □ Heartburn and/or reflux problems  □ Constipation  □ Common cold and/or nasal congestion  □ Pain in the back, neck or pelvic girdle  □ Headache  □ Sleeping problems  □ Others \| \| *If “others”:* **Please indicate which other ailment(s) you have experienced:** \| \| **For each ailment reported, please indicate which medications you have used:** \| |
| --- | --- | --- | --- | --- |

**QUANTIFICATION OF NAUSEA SEVERITY BY THE PUQE SCORE**

During the last 24 hours

| 1. **For how long have you felt nauseated or sick to your stomach?** | | | | |
| --- | --- | --- | --- | --- |
| >6 hours hours **□** | 4-6 hours **□** | 2-3 hours **□** | ≤1 hour **□** | Not at all **□** |
| 1. **How many times have you vomited or thrown up?** | | | | |
| >6 hours hours **□** | 4-6 hours **□** | 2-3 hours **□** | ≤1 hour **□** | Not at all **□** |
| 1. **How many times have you had retching or dry heaves without bringing anything up?** | | | | |
| >6 hours hours **□** | 4-6 hours **□** | 2-3 hours **□** | ≤1 hour **□** | Not at all **□** |

**Thank you for your contribution!**

**The booking form sent to all participants allocated to the intervention group**

| **Your name:** |
| --- |
| **Your telephone phone number:** |
| **Which of the pharmacy do you wish to receive the consultation:**  □ Name and address pharmacy 1  □ Name and address pharmacy 2  □ Name and address pharmacy 3  □ Name and address pharmacy 4  □ Name and address pharmacy 5  □ Name and address pharmacy 6  □ None of the pharmacies above |
| *If one of the pharmacies selected:* **Please specify a date and time you prefer to have the consultation:** |
| *If “None of the pharmacies above”:* If you do not wish to receive the consultation we would like to know why:  □ It is too far to the nearest pharmacy  □ I don’t need a consultation about medications in pregnancy  □ I don’t want to give a reason  □ Other |

**The drop out from available from the e-mails sent to the participants**

| **We would kindly ask you to provide a reason for dropping out of the study:**  □ I am no longer pregnant  □ The questionnaires require too much time  □ I was allocated to the control group  □ The nearest study pharmacy was too far away  □ Other  □ I prefer not to give a reason |
| --- |
