# Supplementary material for: Community pharmacist counseling in early pregnancy—Results from the SafeStart feasibility study
Source: PLoS One. 2019 Jul 19;14(7):e0219424. doi: 10.1371/journal.pone.0219424 (PMC6641474; doi:10.1371/journal.pone.0219424)
Supplement: S2 File — (DOCX) [file pone.0219424.s002.docx]

Pharmacists’ Consultation Guide

The consultation guide provided to all study pharmacists to help structure the consultation. The consultation guide is based on two textbooks in clinical communication [1, 2].

| **Introduction** | Greet the women and introduce yourself. Present yourself as a pharmacist. |
| --- | --- |
|  | Ask the woman what her concerns/needs are, and if she has any specific topics she wants to discuss during this consultation. |
|  | Specify the limits for the consultation, both in regards of time (up to 15 minutes) and content (self-management/pharmaceutical care). |
|  | Based on the woman’s reply, state the topic you wish to address during the consultation. |
| **Mid-part** | Address one topic at the time. |
|  | Focus on mutual understanding; that the woman has understood the information given by you and vice versa. |
|  | Perform the PUQE^a^ assessment, and obtain a PUQE score for all women with symptoms of nausea and/or vomiting. |
|  | Focus on the women´s (prior) experience and perception of the topics. |
| **Closing** | Ask if the woman has any further questions at the end of the consultation |
|  | Ask if she can repeat the main topics discussed/the most important take home messages for her. |
|  | Assess the needs of a follow-up consultation. |

***^a^PUQE*** *= Pregnancy-Unique Quantification of Emesis.* *PUQE-score ≥7 equals moderate or severe NVP.*

References:

1. Frøyland H. Legemiddelsamtalen - og klinisk kommunikasjon [The medicine consultation - and clinical communication]: Fagbokforlaget; 2011.

2. Frøyland H. Klinisk Kommunikasjon [Clinical Communication]. In: Viktil KK, Blix HS, editors. Klinisk farmasi - en lærebok [Clinical pharmacy - a textbook]: Fagbokforlaget; 2017. p. 79-103.
